# Optical insights into spatial precision and release heterogeneity of neuromodulatory transmission

**DOI:** 10.1016/j.isci.2026.116037

**Published:** 2026-05-20

**Authors:** W. Sharon Zheng, Smriti Gupta, Peng Zhang, Yajun Zhang

**Affiliations:** 1 Department of Pharmacology, University of Virginia School of Medicine, Charlottesville, VA 22908, USA; 2 Department of Molecular Physiology and Biological Physics, University of Virginia School of Medicine, Charlottesville, VA 22908, USA; 3 Center for Neurodegenerative Disease and Therapeutics, Rosalind Franklin University of Medicine and Science, North Chicago, IL 60064, USA; 4 Department of Pharmacology and Chemical Biology, Shanghai Jiao Tong University School of Medicine, Shanghai 200025, China

**Keywords:** optical imaging, biological sciences, neuroscience, sensory neuroscience

## Abstract

Intercellular communication via neuromodulatory transmitters, such as serotonin (5-HT) and oxytocin (OXT), underlies a broad range of physiological functions. Although neuromodulatory signaling has traditionally been considered slow, diffuse, and homogeneous volume transmission, accumulating evidence indicates that it can also engage spatially restricted hotspots with high specificity. Studying these modes of transmission has been challenging due to limitations in measuring transmitter release with millisecond precision and single-synapse resolution. Recent advances in imaging and genetically encoded indicators (GEIs) have enabled optical quantal analysis of neuromodulator release with near single-synapse/varicosity precision. In this context, we highlight variability in presynaptic release properties shaping neuromodulatory signaling by decoding quantal size, release probability, readily releasable pool (RRP), and refilling rates—across neuronal subtypes and compartments. We reveal striking heterogeneity in these systems, providing a potential mechanistic basis for how different transmitters, or even a single transmitter, mediate distinct neuromodulatory roles relevant to behavior and function.

## Highlights

• Optical imaging quantifies neuromodulator release with high spatial precision
• Serotonin release exhibits spatial restriction across brain regions and species
• Spatially restricted transmission extends to neuropeptide signaling
• Oxytocin release shows heterogeneous presynaptic properties

## Graphical abstract

**Figure:**
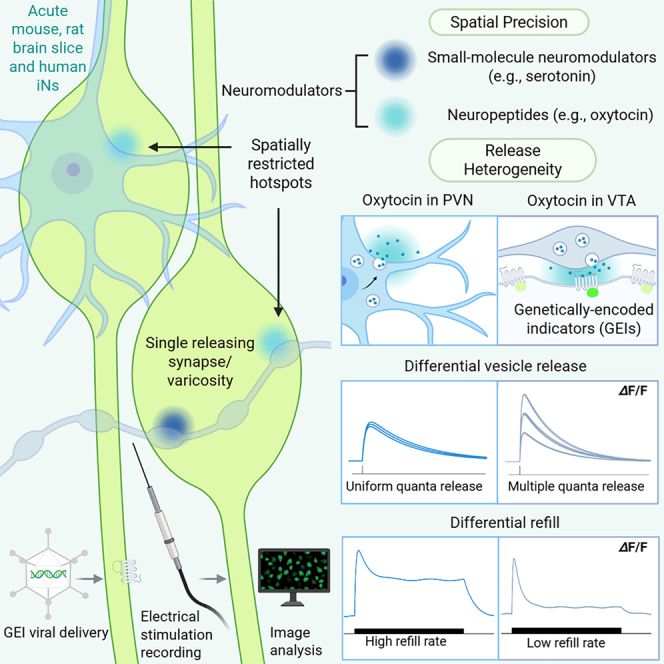

## Introduction

Neuromodulatory transmitters such as serotonin (5-HT) and oxytocin (OXT) have long been thought to mediate slow and diffuse intercellular communication, primarily through volume transmission. However, their critical roles in a wide range of complex and highly coordinated physiological processes, including emotion and social behavior, suggest that they also operate with remarkable speed and spatial precision.^1^^,^^2^^,^^3^^,^^4^ Dysregulation of these systems is closely linked to psychiatric disorders such as depression and autism, further emphasizing their importance in regulating highly coordinated behaviors. Despite this, mechanisms underlying these transmission dynamics remain poorly understood.

Traditionally, volume transmission has been considered the predominant mode for neuromodulators such as 5-HT and OXT, characterized by transmitter diffusion over broad extracellular spaces to reach multiple target cells beyond conventional synaptic contacts.^5^ Yet, emerging evidence extends this classical view, suggesting that neuromodulators may also rely on a more localized spatially restricted “hotspot” transmission mode, characterized by constrained diffusion within synapses/varicosities, producing small, locally confined overlapping domains rather than widespread diffusion.^6^^,^^7^^,^^8^^,^^9^ This paradigm shift is driven by advances in imaging techniques and genetically encoded indicators (GEIs), which now allow high-resolution visualization of neurotransmitter dynamics at the single-synapse level. Utilizing GEIs, we showed that evoked neuromodulator release can occur in a spatially restricted transmission mode.^10^^,^^11^ To further understand the mechanisms and functional relevance of localized transmission, it is essential to characterize key synaptic properties underlying this process. In this study, we achieved high spatiotemporal visualization of 5-HT and OXT release, enabling detailed quantification of critical synaptic properties such as spatial spread length constant, quantal size, quantal content, release probability (Pr), readily releasable pool (RRP) size, and refilling rate at single-synapse resolution. Our findings reveal that presynaptic release properties vary significantly across neuronal subtypes and compartments, reflecting a finely tuned system in which a single neuromodulator can mediate diverse, context-dependent functions. This heterogeneity highlights how different transmitters, or even a single transmitter, mediate overlapping yet distinct roles in neuromodulation, underscoring the conserved yet highly specialized nature of neuromodulatory systems.

## Results

### Pipeline for studying the neuromodulatory transmission properties

**Figure 1:**
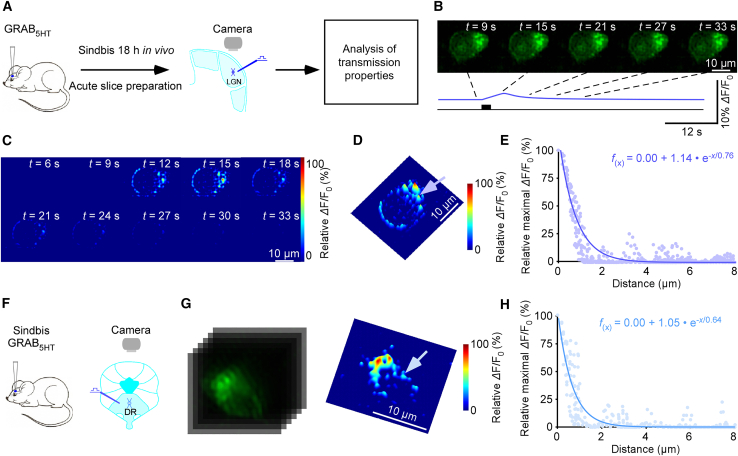
Spatially restricted serotonergic transmission in various brain regions (A) Schematic of stimulation-imaging setup in an *ex vivo* mouse LGN preparation. (B–D) Snapshots (B), heatmaps (C), and 3D spatiotemporal profiling (D) of evoked responses in a geniculate neuron. Light blue arrow indicates an isolated releasing synapse in (D). Scale bars, 10 μm. (E) Pixel-wise maximal *Δ*F/F_0_ at the isolated releasing synapse. Single-exponential fit (light blue line) estimates a spatial spread constant. (F) Schematic of stimulation-imaging setup in an *ex vivo* mouse DR preparation. (G) Snapshots and 3D spatiotemporal profiles of electrically evoked *Δ*F/F_0_ responses in GRAB_5HT1.0_-expressing DR neurons; light blue arrow indicates isolated releasing synapse. Scale bars, 10 μm. (H) Pixel-wise maximal *Δ*F/F_0_ at the isolated releasing synapse. Single-exponential fit (light blue line) estimates a spatial spread constant. See also Figures S1–S9.

To investigate the spatial and temporal dynamics of neuromodulator transmission, we developed a quantitative imaging pipeline based on GEIs. Using GRAB_5HT1.0_ expression in mouse lateral geniculate nucleus (LGN) neurons as an illustrative example, Sindbis virus was injected into the mouse brain, and after an 18 h *in vivo* expression period, acute brain slices were prepared for imaging (Figure 1A). Following 20 pulses of electrical stimulation at 16 Hz with minimal stimulation intensity, fluorescence images were acquired and processed through a multi-step pipeline that included image alignment (Figure S1), baseline correction, denoising, and crucially, deconvolution. The processed images were then used to generate heatmaps and pixel-wise maximal *Δ*F/F_0_ three-dimensional (3D) profiles for analysis of transmission properties. The densely sampled surrounding pixels allowed robust spatial fitting, yielding high-resolution estimates of local diffusion. Meanwhile, *Δ*F/F_0_ responses showed minimal correlation with heterogeneous basal fluorescence F_0_, indicating that signal localization reflects transmitter release rather than sensor expression variability (Figure S9).

We applied deconvolution to correct for optical distortions caused by the point spread function (PSF). We characterized the PSF using fluorescent beads (Figures S2A–S2C). Mean square error stabilized and signal-to-noise ratio plateaued after ∼25 iterations (Figures S2D and S2E), indicating robust convergence of the deconvolution process. The pixel-wise 3D profile of maximal fluorescence intensity (*Δ*F/F_0_) showed that deconvolution revealed a steeper spatial decay with distance from the center of the release spot (Figure S2F). We quantified the spread of the neuromodulatory signal by fitting the spatial decay of *Δ*F/F_0_ and calculating the spread length constant. Without deconvolution, the spread length constant of the indicated synapse was approximately 1.01 μm, indicating a broader apparent diffusion. After deconvolution, the spread length constant decreased to approximately 0.74 μm, suggesting a more localized signal transmission (Figure S2G). This reduction was statistically significant, underscoring the importance of deconvolution for accurate measurement of transmission properties.

Time-lapse imaging further illustrated the dynamic process of neuromodulator release and spread. Raw fluorescent images over time (Figure 1B) and corresponding heatmaps of relative ΔF/F_0_ (Figure 1C) showed the gradual emergence and dissipation of the signal following stimulation, enabling the assessment of both the spatial and temporal aspects of neuromodulatory transmission. To rule out the possibility that spatially restricted ΔF/F_0_ signals arise from heterogeneous sensor expression or sampling geometry, we performed an exogenous serotonin puff application as a control. A low-pressure puff with extended duration was applied to generate a relatively broad extracellular serotonin field distinct from electrical stimulation. When compared with electrically evoked responses in the same neuron, puff application produced more spatially diffuse and less punctate fluorescence patterns (Figure S3). These results indicate that the response hotspots observed during electrical stimulation are not attributable to heterogeneous sensor accessibility but reflect spatially restricted transmitter release. Together, we establish a robust framework for quantifying neuromodulator transmission properties with high spatial and temporal precision.

### Spatially restricted serotonergic transmission in various brain regions and species

**Figure 2:**
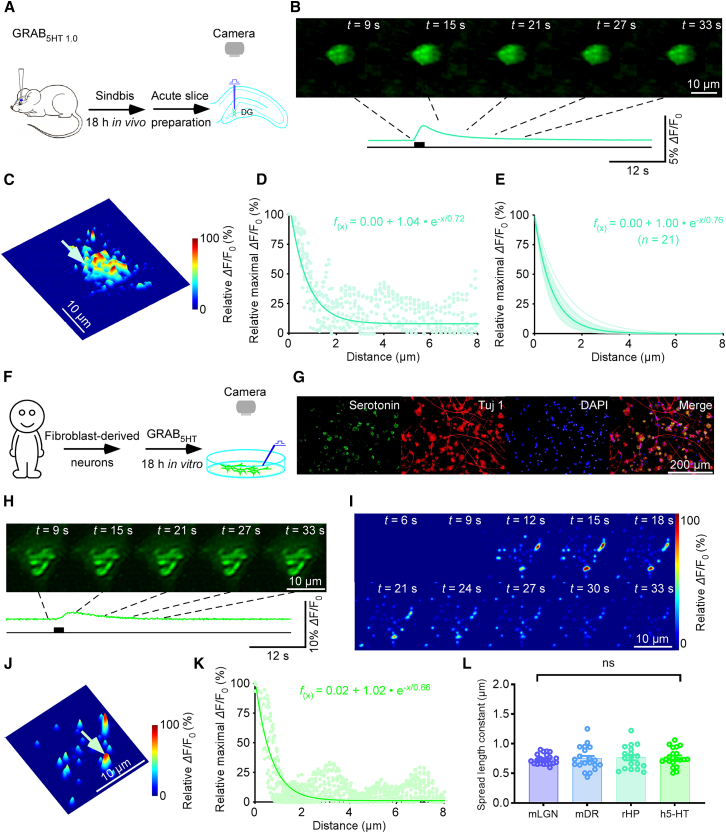
Spatially restricted serotonergic transmission in various species (A) Schematic of stimulation-imaging experiment in an *ex vivo* rat HP preparation. (B and C) Snapshots (B), 3D spatiotemporal profiling (C) of evoked responses in a DG neuron. Green arrow indicates an isolated releasing synapse in (C). Scale bars, 10 μm. (D) Pixel-wise maximal *Δ*F/F_0_ at the isolated releasing synapse. Single-exponential fit (green line) estimates a spatial spread constant. (E) Summary of 5-HT diffusion curves from putative single releasing synapses (spread length constant: 0.76 ± 0.04; *n* = 21 synapses from 7 neurons, average curve in dark green). (F) Schematic of stimulation-imaging experiment in a human fibroblast-derived neuron culture preparation. (G) Immunostaining of human fibroblast-derived serotonergic neurons. Green, anti-5-HT staining; red, Tuj1 anti-tubulin *β*3 staining; blue: DAPI. Scale bars, 200 μm. (H–J) Snapshots (H), heatmaps (I), and 3D spatiotemporal profiling (J) of evoked *Δ*F/F_0_ responses in a GRAB_5HT1.0_ expressing human fibroblast-derived neuron; blue arrow indicates isolated releasing synapse in (J). Scale bars, 10 μm. (K) Pixel-wise maximal *Δ*F/F_0_ at the isolated releasing synapse. Single-exponential fit (light green line) estimates a spatial spread constant. (L) Spread length constants for 5-HT across species and brain regions: mouse LGN neurons (0.74 ± 0.02, *n* = 22 synapses from 8 neurons), mouse DR neurons (0.68 ± 0.03, *n* = 20 synapses from 11 neurons), rat HP neurons (0.76 ± 0.04, *n* = 21 synapses from 7 neurons), and human fibroblast-derived neurons (0.70 ± 0.03, *n* = 20 synapses from 11 neurons); ns, no significant differences were detected among groups, Kruskal-Wallis test, *p* = 0.27, effect size *ε*^2^ = 0.012, indicating a negligible between-group effect. Data are represented as mean ± SEM. See also Figures S5 and S9.

Building on our validated imaging pipeline, we next applied this approach to study serotonergic transmission across different brain regions and species using GRAB_5HT1.0_. By visualizing fluorescence responses at discrete transmitter-releasing synapses, we characterized key properties of 5-HT signaling at single-synapse resolution. In the mouse LGN, a well-characterized visual relay center that receives dense serotonergic innervation,^12^ electric stimulation with 20 pulses at 16 Hz evoked 5-HT release events (Figures 1B and 1C). Fitting a single-exponential decay to the pixel-wise maximal *Δ*F/F_0_ 3D profile yielded a 5-HT spread length constant of 0.74 μm at the indicated synapse (Figures 1D and 1E). Bath application of tetrodotoxin (TTX), which blocks action potential-dependent synaptic transmission, abolished evoked responses in GRAB_5HT1.0_-expressing LGN neurons (Figure S4), confirming their synaptic origin. These findings suggest that evoked 5-HT release in the LGN occurs via a spatially restricted transmission mode. To assess the generality of this transmission mode, we extended our analysis to other brain regions and species. In mouse dorsal raphe nucleus (DR), where serotonergic neurons originate, Sindbis-mediated GRAB_5HT1.0_ expression and acute slice preparation enabled visualization of 5-HT release events, yielding a spread length constant of 0.64 μm at the indicated peak (Figures 1F–1H). Applying the same approach to an *ex vivo* rat hippocampal preparation, we observed serotonergic responses in dentate gyrus (DG) neurons with a spread length constant of approximately 0.76 μm (Figures 2A–2E).

We further examined serotonergic transmission in human neurons using an established human fibroblast-derived serotonergic neuron model^13^ (Figures 2F and 2G). GRAB_5HT1.0_ expression and local stimulation with 20 pulses at 16 Hz evoked fluorescence responses (Figures 2H and 2I), with a spread length constant of ∼0.66 μm at the indicated peak (Figures 2J and 2K). Across all examined systems, including mouse LGN and DR, rat HP, and human fibroblast-derived serotonergic neurons, the measured 5-HT spread length constants were statistically indistinguishable, averaging around 0.70 μm (Figure 2L). Notably, within the same region, the estimated spread length constant was largely independent of sensor expression level (Figure S5). This consistency across species and brain regions supports the notion that spatially restricted transmission may represent a reproducible feature of serotonergic signaling.

### Analysis of the presynaptic release properties of serotonergic transmission

**Figure 3:**
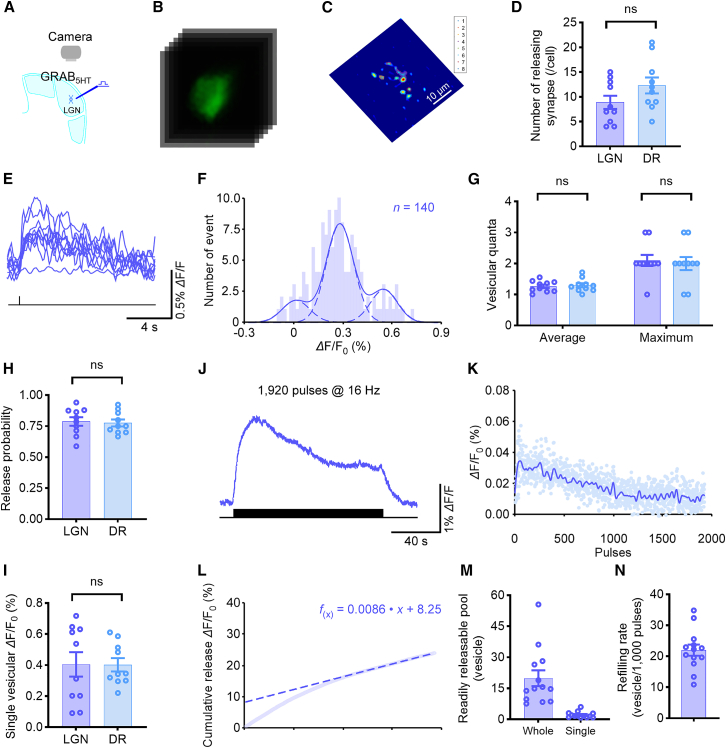
Analysis of the presynaptic release properties of serotonergic transmission (A) Schematic of stimulation-imaging setup in an *ex vivo* mouse LGN preparation. (B) Snapshots of electrically evoked *Δ*F/F_0_ responses in GRAB_5HT1.0_-expressing LGN neurons. (C) Schematic illustration of the identification of the releasing synapses. Scale bars, 10 μm. (D) Average number of releasing synapses per neuron (LGN, 8.9 ± 1.3; *n* = 10 neurons; DR, 12.3 ± 1.62; *n* = 10 neurons, *r* = −0.35, 95% CI [−0.83, 0.07]); ns, no significant differences, *p* > 0.05, Mann-Whitney rank-sum tests. (E) Ten single-pulse evoked *Δ*F/F_0_ responses at the isolated releasing synapse. (F) Amplitude histograms of *Δ*F/F_0_ responses showing multiple, evenly spaced peaks. (G–I) Average (LGN, 1.26 ± 0.06; DR, 1.29 ± 0.07, *r* = −0.15, 95% CI [−0.66, 0.35]) and maximal (LGN, 2.10 ± 0.18; DR, 2.00 ± 0.21, *r* = 0.00, 95% CI [−0.40, 0.39]) vesicular quanta, release probability (LGN, 0.79 ± 0.03; DR, 0.78 ± 0.03, *r* = −0.29, 95% CI [−0.77, 0.15]), and quantal size *Δ*F/F_0_ (LGN, 0.40% ± 0.08%; DR, 0.43% ± 0.04%, *r* = −0.84, 95% CI [−1, 1]) at single 5-HT releasing synapses; ns, no significant differences, *p* > 0.05, Mann-Whitney rank-sum tests. (J) ΔF/F_0_ responses evoked by 1,920 local electrical stimuli at 16 Hz. (K) The *Δ*F/F_0_ response to each individual stimulus. (L) Cumulative *Δ*F/F_0_ plot versus stimulus number; RRP size and refilling rate inferred from the *y* intercept and slope of a linear fit to late points. (M and N) RRP (whole cell, 19.93 ± 3.82 vesicles; single releasing synapse, 2.17 ± 0.41 vesicles; *n* = 13 neurons, effect size *r* = −0.84) and vesicular refilling rate (22.0 ± 0.2 vesicles per 1,000 pulses; *n* = 13 neurons) of GRAB_5HT1.0_-expressing geniculate neurons. Data are represented as mean ± SEM. See also Figure S9.

Having shown that serotonergic transmission displays spatially restricted signaling, we next sought to dissect the presynaptic properties underlying 5-HT release by leveraging our imaging approach. Direct visualization of 5-HT release at isolated subcellular sites provided a powerful platform to quantify presynaptic properties in both the LGN, which receives dense serotonergic projections, and the DR, where 5-HT neurons originate. In both regions, GRAB_5HT1.0_-expressing neurons exhibited around 10 evoked releasing synapses, identified using the density-based spatial clustering algorithm (Figures 3A–3D). We then examined *Δ*F/F_0_ responses at single releasing synapses of GRAB_5HT1.0_-expressing neurons, evoked by locally delivered trains of electric pulses at 0.1 Hz. These responses appeared as stochastic events, with a mixture of failures and successful *Δ*F/F_0_ events of varying amplitudes in LGN (Figure 3E). A histogram of *Δ*F/F_0_ amplitudes in LGN, inspired by classical quantal analysis,^14^ revealed multiple, approximately equally spaced peaks, consistent with quantized vesicular release (Figure 3F). On average, each stimulus evoked ∼1.25 vesicular quanta, with up to 3 quanta observed per event (Figure 3G) for both LGN and DR neurons. This quantal analysis further enabled determination of a Pr of ∼0.75 and a single-vesicle quantal size of approximately 0.40% *Δ*F/F_0_ at individual serotonergic releasing synapses for both LGN and DR neurons (Figures 3H and 3I). These results indicate that LGN and DR synapses exhibit similar presynaptic release properties, including quantal size, Pr, and synapse number.

To further characterize presynaptic release properties, we delivered a prolonged stimulus consisting of 1,920 pulses at 16 Hz to GRAB_5HT1.0_-expressing geniculate neurons. Initially, large *Δ*F/F_0_ responses were observed, which gradually plateaued during continued stimulation (Figure 3J). Fluorescence *Δ*F/F_0_ responses of individual release events used in the plots were calculated by mathematically correcting for the effects caused by the off-rates of GRAB_5HT1.0_ sensors (τ_off_ = 8.96 s) (Figure 3K). Cumulative release plots were generated and fitted based on a synaptic transmission model,^15^ enabling estimation of RRP and vesicle refilling rate (Figure 3L). Specifically, back-extrapolation of the linear fit to the late phase of the cumulative trace provided the size of the RRP, while the slope of the fit indicated the vesicle refilling rate.^16^ Converting *Δ*F/F_0_ values to quantal size revealed an RRP of approximately 20 vesicles per neuron and ∼2 vesicles per individual releasing synapse (Figure 3M). The estimated vesicle refilling rate was approximately 20 vesicles per 1,000 stimuli (Figure 3N). Collectively, these results demonstrate that imaging of 5-HT release enables precise quantification of key presynaptic properties, including the number of releasing synapses, quantal size, quantal content, Pr, RRP size, and vesicle refilling kinetics in serotonergic neurons.

### Heterogeneity of OXT synapses release properties

**Figure 4:**
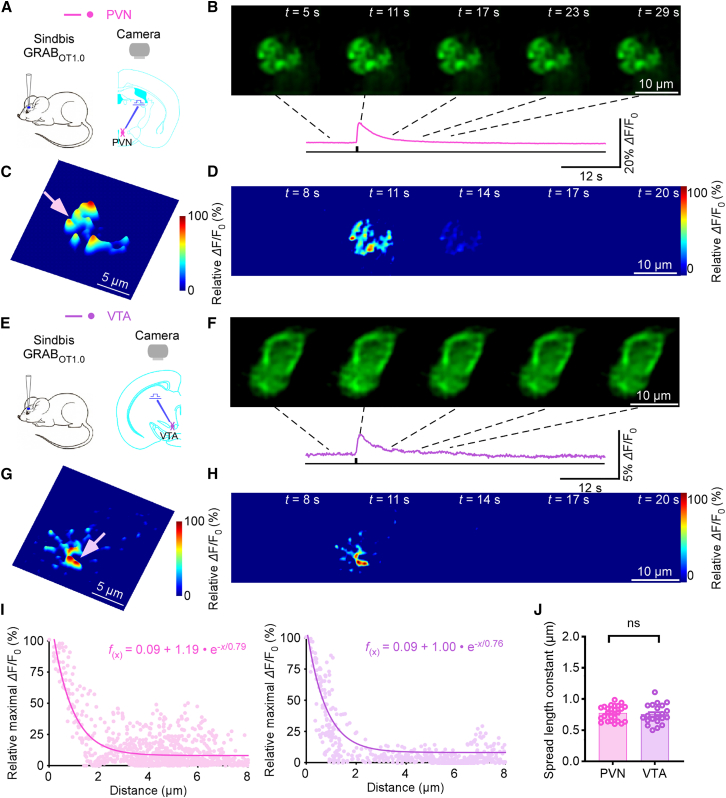
Spatially restricted OXTergic transmission in various brain regions (A) Schematic of stimulation-imaging experiment in an *ex vivo* PVN preparation. (B–D) Snapshots (B), 3D spatiotemporal profiling (C) and heatmaps (D) of evoked responses (64 Hz, 10 pulses) in a PVN neuron. Scale bars, 10 μm. Pink arrow indicates an isolated releasing synapse in (C). Scale bars, 5 μm. (E) Schematic of stimulation-imaging experiment in an *ex vivo* VTA preparation. (F–H) Snapshots (F), 3D spatiotemporal profiling (G) and heatmaps (H) of evoked responses (64 Hz, 10 pulses) in a VTA neuron. Scale bars, 10 μm. Pink arrow indicates an isolated releasing synapse in (H). Scale bars, 5 μm. (I) Pixel-wise maximal *Δ*F/F_0_ at the isolated releasing synapse. Single-exponential fit (PVN, pink line; VTA: purple line) estimates a spatial spread constant. (J) Spread length constants for OXT (PVN. 0.78 ± 0.02 μm, *n* = 25 synapses from 10 neurons; VTA, 0.75 ± 0.03, *n* = 21 synapses from 8 neurons; *U* = 281.0, *p* = 0.69; ns, no significant differences, *r* = 0.058, 95% CI [−0.28, 0.41], Mann-Whitney rank-sum tests). Data are represented as mean ± SEM. See also Figure S9.

Having established an analytical framework for serotonergic transmission, we next asked whether similar principles apply to neuropeptide signaling, focusing on OXT—a multifunctional neuromodulator also involved in diverse emotion and social behavior.^17^ To examine OXTergic transmission, we expressed the genetically encoded OXT sensor GRAB_OT1.0_^18^ in the mouse paraventricular nucleus (PVN) and ventral tegmental area (VTA) ex *vivo*. The PVN is where OXT neurons originate, while the VTA receives OXT projections.^19^^,^^20^ After 18 h, acute brain slices were prepared, and electrically evoked OXT fluorescence responses by 10 pulses at 64 Hz were recorded (Figures 4A–4H). Spatial analysis revealed highly restricted OXT diffusion, with a spread length constant of ∼0.75 μm in both regions (Figures 4I and 4J), suggesting that OXT, like 5-HT, undergoes spatially restricted synaptic transmission.

**Figure 5:**
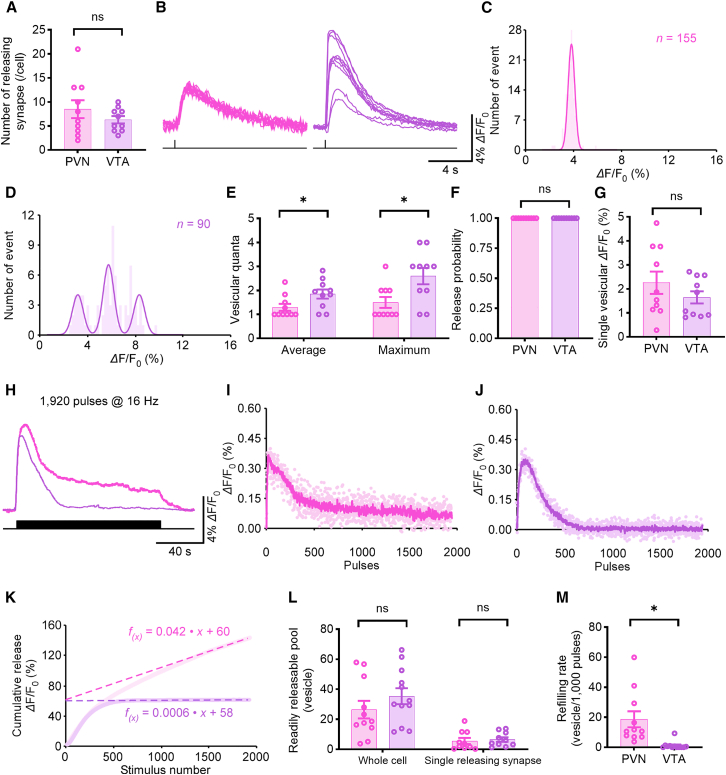
Heterogeneity of OXT synapses release properties (A) Average number of releasing synapses of PVN and VTA neurons (PVN, 8.5 ± 1.9, *n* = 10 neurons; VTA, 6.3 ± 0.8; *n* = 10 neurons; *U* = 42.0, *p* = 0.57, *r* = 0.12, 95% CI [−0.38, 0.66]); ns, no significant differences, Mann-Whitney rank-sum tests. (B) Ten *Δ*F/F_0_ responses evoked by single-pulse stimuli at the single releasing synapse in PVN and VTA. (C and D) Amplitude histograms of *Δ*F/F_0_ responses showed nearly equal peaks for PVN and multiple (C), nearly equally spaced peaks for VTA (D). (E–G) Values for the average (PVN, 1.30 ± 0.15, *n* = 10 neurons; VTA, 1.85 ± 0.19, *n* = 10 neurons; *U* = 76, *p* < 0.05, *r* = −0.44, 95% CI [−0.06, −0.89]) and maximal (PVN, 1.5 ± 0.22, *n* = 10 neurons; VTA, 2.60 ± 0.34, *n* = 10 neurons; *U* = 79.0, *p* < 0.05, *r* = −0.50, 95% CI [−0.91, −0.15]) vesicular quanta, release probability (PVN, 100.0% ± 0.0%, *n* = 10 neurons; VTA, 100.0% ± 0.0%, *n* = 10 neurons; *U* = 50.0, *p* < 0.001), and quantal size (PVN, 2.26% ± 0.46% *Δ*F/F_0_, *n* = 10 neurons; VTA, 1.65% ± 0.25% *Δ*F/F_0_, *n* = 10 neurons; *U* = 41.0, *p* = 0.52, *r* = 0.26, 95% CI [−0.22, 0.76]) at the single releasing synapse for PVN and VTA neurons. (H) *Δ*F/F_0_ responses of GRAB_OT1.0_ expressing PVN and VTA neuron evoked by local electrical stimuli of 1,920 pulses at 16 Hz. (I and J) The *Δ*F/F_0_ response to each individual stimulus. (K) Plots of cumulative *Δ*F/F_0_ responses against the stimulus number in GRAB_OT1.0_ expressing PVN and VTA neurons. Note the RRP size and refilling rate inferred from the *y* axis intercept and slope of linear fit to late points of the cumulative trace, respectively. (L and M) Values for the RRP (PVN, 26.4 ± 5.8, *n* = 11 neurons; VTA, 35.4 ± 5.3, *n* = 12 neurons; *U* = 85.0, *p* = 0.26, *r* = −0.23, 95% CI [−0.74, 0.21] for whole cells; PVN, 5.57 ± 2.0, *n* = 11 neurons; VTA, 6.54 ± 1.49; *n* = 12 neurons; *U* = 71.0, *p* = 0.121, ns, no significant differences, *r* = −0.17, 95% CI [−0.70, 0.32] for single releasing synapses) and vesicular refilling rate (PVN, 18.64 ± 5.32 vesicles per 1,000 pulses, *n* = 11 neurons; VTA, 1.12 ± 0.75 vesicles per 1,000 pulses, *n* = 12 neurons; *U* = 4.0, ∗*p* < 0.001 with significant differences, *r* = −0.79, 95% CI [−0.77, −1]) of GRAB_OT1.0_ expressing PVN and VTA neurons. Data are represented as mean ± SEM. See also Figures S6 and S8.

We then characterized the presynaptic properties of OXTergic synapses. We detected discrete OXT release events at ∼8.5 synapses per GRAB_OT1.0_-expressing neuron in the PVN and ∼6.3 synapses in the VTA, a difference that was not statistically significant (Figure 5A). Trains of stimulation at 0.1 Hz evoked stochastic *Δ*F/F_0_ responses at single releasing synapses in both regions (Figure 5B). *Δ*F/F_0_ amplitude histograms again showed discrete, evenly spaced peaks indicative of quantized release in PVN and VTA (Figures 5C and 5D). On average, each stimulus evoked ∼1.30 vesicular quanta in PVN and ∼1.85 in VTA, with VTA also exhibiting a higher maximum of up to 4 quanta per event (Figure 5E). Further analysis revealed that multivesicular release peaks sum approximately linearly (Figure S6A), and peak areas of deconvolved single events also exhibit a quantized distribution consistent with linear summation (Figure S6B). Despite this difference in vesicular quanta, the Pr at individual synapses approached 1.0 in both regions (Figure 5F), and quantal size was comparable with no significant difference (Figure 5G).

Upon prolonged high-frequency stimulation (1,920 pulses at 16 Hz), cumulative release analysis revealed distinct synaptic dynamics between brain regions. Releasing synapses at PVN maintained more stable OXT release, whereas synapses at VTA exhibited significant rundown of the fluorescence signal (Figure 5H). Fluorescence *Δ*F/F_0_ responses of individual release events used in the plots were calculated by mathematically correcting for the effects caused by the slow off-rates of GRAB_OT1.0_ sensors (τ_off_ = 3.15 s) (Figures 5I and 5J). Linear fitting of cumulative release plots (Figure 5K) showed that while RRP sizes were similar between PVN and VTA neurons (Figure 5L), the vesicle refilling rate was significantly lower in VTA neurons (∼1 vesicle/1,000 pulses) compared to PVN neurons (∼20 vesicles/1,000 pulses) (Figure 5M). These findings demonstrate that OXTergic transmission, like 5-HT, shows spatially restricted transmission across brain regions, but exhibits region-specific heterogeneity in presynaptic release properties—reflecting potential adaptations to distinct circuit functions.

### Spatial properties of transmission across diverse neuromodulators

**Figure 6:**
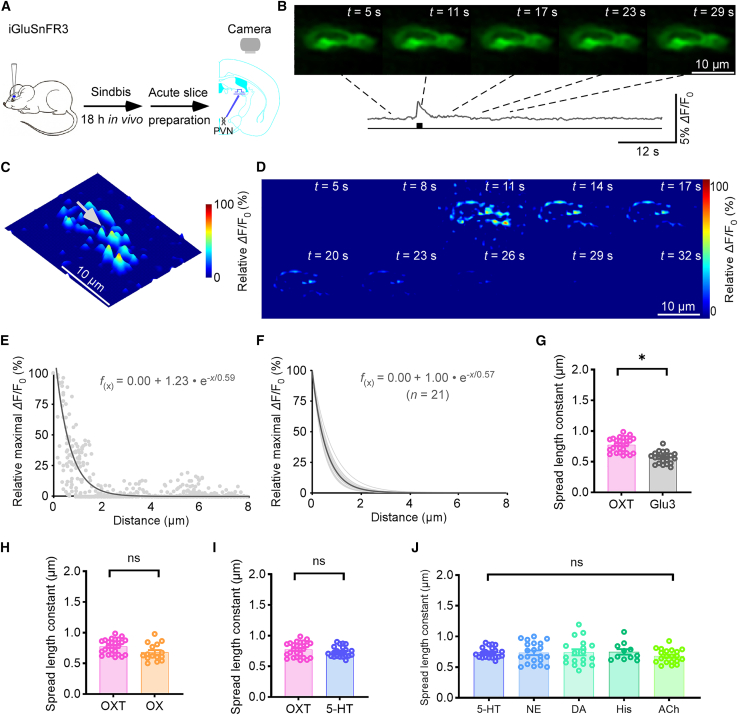
Spatial properties of transmission across diverse neuromodulators (A) Schematic of stimulation-imaging experiment in an *ex vivo* PVN preparation. (B–D) Snapshots (B), 3D spatiotemporal profiling (C)**,** heatmaps (D) electrically evoked responses in an iGluSnFR3 expressing PVN neuron; gray arrow indicates an isolated releasing synapse. Scale bars, 10 μm. (E) Pixel-wise maximal *Δ*F/F_0_ at the isolated releasing synapse. Single-exponential fit (gray line) estimates a spatial spread constant. (F) Summary of glutamate diffusion curves from putative single releasing synapses (spread length constant: 0.57 ± 0.02; *n* = 15 synapses from 9 neurons, average curve in dark gray). (G) Spread length constants for OXT and glutamate at PVN (OXT, 0.78 ± 0.02 μm, *n* = 25 synapses from 10 neurons; Glu3, 0.57 ± 0.03, *n* = 21 synapses from 8 neurons; *U* = 42, ∗*p* < 0.001 with significant differences, *r* = 0.72, 95% CI [0.96, 0.66], Mann-Whitney rank-sum tests). (H) Spread length constants for OXT and OX (OXT, 0.78 ± 0.02 μm, *n* = 25 synapses from 10 neurons; OX, 0.68 ± 0.04, *n* = 15 synapses from 6 neurons; *U* = 102.5, *p* > 0.05, ns, no significant differences, *r* = 0.32, 95% CI [0.73, 0.03], Mann-Whitney rank-sum tests). (I) Spread length constants for OXT and 5-HT (OXT, 0.78 ± 0.03 μm, *n* synapses = 25 from 10 neurons; 5-HT, 0.75 ± 0.03, *n* synapses = 22 from 8 neurons; *U* = 230.5, *p* > 0.05, ns, no significant differences, *r* = 0.12, 95% CI [0.48, −0.18], Mann-Whitney rank-sum tests). (J) Spread length constants for various transmitters: 5-HT at LGN (0.74 ± 0.02, *n* synapses = 22 from 8 neurons), NE at basolateral amygdala (BLA) (0.67 ± 0.03, *n* = 20 synapses from 10 neurons), DA at caudate putamen (CPu) (0.73 ± 0.05, *n* = 20 synapses from 10 neurons), His at BLA (0.75 ± 0.04, *n* = 11 synapses from 6 neurons), ACh at BLA (0.68 ± 0.02, *n* = 21 synapses from 10 neurons); ns, no significant differences were detected among groups (Kruskal-Wallis test, *p* = 0.17, effect size *ε*^2^ = 0.025, indicating a negligible between-group effect). Data are represented as mean ± SEM. See also Figures S7–S9.

To assess whether spatially restricted transmission is a general feature across neurotransmitter systems examined here, we extended our analysis to include multiple classical and neuromodulatory transmitters. We first examined glutamate release using iGluSnFR3-V857^21^ expressed in the PVN, the same region used to study OXTergic transmission, allowing for direct comparison (Figure 6A). Local electrical stimulation using 20 pulses at 64 Hz evoked fluorescence increases at discrete subcellular locations on the soma (Figure 6B). Time-lapse heatmaps and 3D projections revealed multiple spatially restricted releasing synapses (Figures 6C and 6D). Spatial decay of the *Δ*F/F_0_ signal was fit to a single-exponential function, yielding a spread length constant of ∼0.59 μm in representative cells (Figure 6E) at the indicated synapse. Across 21 synapses, the average spread length constant was ∼0.57 μm (Figure 6F), which was significantly smaller than that observed for OXT release suggesting relatively more localized glutamatergic transmission (Figure 6G). As a control, similar glutamate spread estimates were obtained using another iGluSnFR A184V variant with different affinity and dynamic range, suggesting that this difference is unlikely to arise from sensor characteristics (Figure S7).

To further generalize these findings, we investigated another neuropeptide orexin (OX) using the GEI OxLight1,^22^ expressed in the nucleus accumbens shell (NAcSh) (Figure S8A). Evoked OX release with 20 pulses at 64 Hz resulted in punctate fluorescence increases with minimal baseline signal (Figure S8B). 3D visualization and time-resolved heatmaps showed clear, restricted release events (Figures S8C and S8D). The spatial profile of orexinergic transmission yielded a spread length constant of ∼0.67 μm at the indicated individual synapse (Figure S8E), and the average spread length constant across 15 synapses was ∼0.68 μm (Figure S8F), with no significant difference from OXT (Figure 6H), supporting restricted transmission.

Finally, to compare spatial transmission across systems, we compiled the spread length constants across all neuromodulators examined. This analysis included the neuropeptide OXT and the monoamine 5-HT (Figure 6I), as well as additional monoaminergic and modulatory transmitters, including norepinephrine (NE) by GRAB_NE1m_, dopamine (DA) by GRAB_DA2m_, histamine (His) by GRAB_HA1m_, and acetylcholine (ACh) by iAChSnFR evoked by 20 pulses at 16 Hz (Figure 6J). Across all neuromodulators analyzed, spread length constants appeared to cluster within a relatively narrow range under the specific stimulation and imaging conditions used for each transmitter-sensor pair. Transmitter signals exhibited spatially restricted profiles rather than broad, homogeneous diffusion. Together, our findings indicate that spatially restricted neuromodulator signals can be detected across diverse molecules, neuron types, and brain regions.

## Discussion

Our findings refine the prevailing view of neuromodulatory transmission by demonstrating that both monoamines and neuropeptides can engage in spatiotemporally precise release while also relying on slow diffusion. Leveraging GEIs and high-resolution imaging, we uncovered synaptic features—such as quantal size, Pr, vesicle pool size, and diffusion range—for transmitters like 5-HT and OXT. We identified heterogeneity in the synaptic properties of OXTergic synapses, particularly in release pool size. These results highlight the complexity and fine-tuned control underlying transmitter release in regulating circuit function and behavior.

### Restricted transmission in monoamines and peptides

Neuromodulatory transmission is increasingly recognized as more complex than the classical view of slow, homogeneous diffusion.^23^ Our study extends the classical view by showing that neuromodulatory transmitters need not operate solely through diffuse signaling and influence broad regions over extended timescales. Using high-resolution optical tools, we showed that monoamines (5-HT, NE, and DA) and neuropeptides (OXT, OX) can exhibit spatially restricted and temporally precise release patterns characteristic of synaptic transmission. We demonstrate that both monoamines and neuropeptides are detected at discrete presynaptic sites with tightly regulated temporal dynamics, despite their release from structurally distinct vesicle types—large dense-core vesicles (LDCVs) for neuropeptides^24^ and small synaptic vesicles (SSVs) for monoamines. Notably, their local diffusion profiles were comparable, indicating similar spatial precision.

Spatially restricted release enables neuromodulatory neurons to encode firing patterns into distinct transmission modes. Our previous work revealed that firing patterns and transporter gating determine whether serotonin signaling remains localized or shifts toward spillover, supporting a tunable, frequency-dependent regulation.^11^ Current stimulation protocols (pulse number and frequency) were empirically optimized for each transmitter to achieve reliable, non-saturating *Δ*F/F_0_ responses with minimal stimulation. These differences may affect the absolute values and thus limit strict quantitative equivalence across transmitter systems. Future studies employing standardized stimulation protocols across transmitter systems will further strengthen direct cross-system comparisons. Although conducted in acute slices, our experiments were performed in highly viable preparations that closely mimic physiological conditions, indicating that the observed localization reflects intrinsic presynaptic properties. By focusing on imaging somatic synapses, where sensor expression and optical access ensured high signal-to-noise and reproducible quantification, our approach enabled precise characterization of neuromodulator release spread under controlled conditions. The consistent spread lengths and hotspot localization across cells and regions further support intrinsic confinement rather than sampling bias. This analytical framework can be extended to imaging of dendritic synapses as well.^11^

### Rethinking somatodendritic versus axonal release

Our results prompt a critical reassessment of the long-held dichotomy between somatodendritic and axonal release. While it is commonly believed that neuromodulator release occurring near the neuronal soma is primarily mediated by dendritic mechanisms,^25^ this view may not fully reflect the anatomical and functional complexity of transmitter release. This perspective has shaped much of our understanding of neural signaling, with axonal release considered to be largely confined to distal projections. Our data suggest that this assumption may be overly simplistic and may not fully capture the complexity of neurotransmitter dynamics in soma-proximal regions. We provide evidence that local axon collaterals can contribute significantly to transmitter release in soma-proximal regions. For instance, our experiments demonstrated that 5-HT showed similar presynaptic release properties in both the LGN and DR, suggesting conserved mechanisms that are not strictly compartment-dependent. These findings are consistent with emerging evidence^7^ and challenge the intuition that perisomatic release must originate from dendrites—it may, in fact, originate from locally projecting axons. Recognizing the role of local axon collaterals allows for a more accurate model of neuromodulator signaling in the brain.

### Heterogeneity of peptide release within single modulatory neuron type

In addition to highlighting the important role of collateral axonal release near the soma, our findings reveal heterogeneity in release properties—particularly for neuropeptides—within a single modulatory neuron type. OXT release is not uniform; instead, it varies depending on the location of the synapse and the specific neuronal subtype involved. Specifically, OXT-releasing synapses exhibited distinct presynaptic characteristics in the PVN and VTA, consistent with previous findings that compartment-specific release is governed by different molecular machinery and calcium channel dependencies.^18^

In addition to subcellular compartmentalization, neuronal subtype identity provides an independent source of heterogeneity. Notably, we also identified substantial variability within the same brain region. Both PVN and VTA contained outlier synaptic clusters that deviated from the dominant local profile. In the PVN, OXT neurons comprise both magnocellular and parvocellular neurons.^26^ Magnocellular neurons are classically defined by their projections to the posterior pituitary but also send axon collaterals to multiple forebrain regions,^27^^,^^28^ whereas parvocellular neurons send axonal projections to the whole brain.^29^ The presence of divergent synaptic clusters in the VTA and PVN may reflect contributions from distinct OXT neuron subtypes rather than solely axonal–dendritic or compartmental differences. Consistent with recent evidence of morpho-electric and transcriptomic diversity within PVN neurons,^30^ these findings support a multidimensional model of neuron identity shaped by subcellular specialization, projection patterns, and functional output.

Because OXT is a neuropeptide that lacks rapid reuptake, replenishment of releasable vesicles primarily depends on peptide synthesis and axonal transport rather than fast local recycling, in contrast to classical small-molecule neurotransmitters. The similar RRP sizes but divergent refilling rates in PVN and VTA terminals may therefore arise from multiple, not mutually exclusive mechanisms, including differences in Ca^2+^ handling, or the composition of OXT neuron populations innervating these regions.^19^^,^^27^ Future experiments manipulating Ca^2+^ conditions or identifying OXT neuron subtypes of PVN and VTA terminals will be important to test these hypotheses. Together, the spatial heterogeneity likely enables OXT neurons to exert precise and context-dependent modulation across diverse target circuits involved in social bonding, feeding, and stress responses.^31^

Beyond these physiological insights, our study introduces several key conceptual and methodological advances. By applying an identical imaging and analytical pipeline to both serotonin and OXT, we enable direct cross-transmitter comparison under identical conditions. This framework reveals that spatially confined release is a shared yet heterogeneously tuned feature across transmitter types. Additionally, integrating quantitative modeling enabled estimation of presynaptic properties from optical data, complementing electrophysiological approaches. Enhanced spatial deconvolution and automated synapse detection improved reproducibility and facilitated cross-regional mapping of neuromodulatory release. Because the sensors used differ in affinity, dynamic range, and kinetic characteristics (Table S1), we explicitly considered potential measurement biases. Spatial diffusion profiles were derived from the maximal *Δ*F/F_0_ reached at each pixel across time, rather than from time-integrated signals, making diffusion length estimates largely insensitive to differences in sensor kinetics. Although slower kinetics could influence long-term stimulation recordings, such effects are minimized by sensor-specific kinetic correction and are unlikely to impact comparisons made using the same sensor for a given modulator. In contrast, when comparing presynaptic properties across different neuromodulators, differences in sensor characteristics remain an important consideration. Collectively, these advances establish a generalizable framework for studying neuromodulator signaling with single-event precision across multiple transmitter systems.

### Implications for disease and therapy

Our findings reveal that neuromodulatory transmission operates with remarkable spatial and temporal precision, challenging the classical notion of slow, homogeneous signaling. This paradigm shift has important implications for neuropsychiatric disorders such as depression, anxiety, and autism, where pathology may stem not from global transmitter deficits but from disrupted compartment-specific release dynamics. A deeper understanding of the molecular machinery governing synaptic versus extrasynaptic release could inform the development of more targeted therapeutic strategies. In this context, recognizing the fine-tuned control of neuromodulator release opens the door to precision interventions at the circuit and compartment level, potentially offering greater efficacy with fewer side effects.^32^

To explore the translational relevance of our findings, we employed fibroblast-derived serotonergic neurons and propose expanding this approach to human induced pluripotent stem cell (iPSC)-derived cholinergic and dopaminergic neurons. These platforms allow for the direct study of human-specific synaptic properties and disease-associated alterations in neuromodulator release. Importantly, iPSC-derived neurons retain the intrinsic release machinery and closely replicate native neuronal physiology.^33^ When derived from individuals with neuropsychiatric disorders, these models preserve patient-specific genetic backgrounds, enabling cellular-level investigations of disease phenotypes and personalized drug responses. Integrating high-resolution imaging and GEIs with human iPSCs-derived neurons offers a powerful platform for precision medicine by enabling targeted drug screening, regenerative therapies, and the investigation of diverse neuromodulatory signaling in health and disease. Altogether, our work highlights the potential of combining mechanistic insights with human-based models to deepen our understanding of brain function and advance precision therapies for neurological and psychiatric disease.^34^

### Limitations of the study

To achieve high temporal resolution, our experiments used wide-field epifluorescence imaging in relatively thick acute brain slices. Although deconvolution improved lateral spatial precision, fluorescence signals from different axial planes may still overlap, potentially leading to underestimation of true spatial spread. Future studies combining post-fixation super-resolution imaging or two-photon microscopy will be important for resolving the full 3D organization of neuromodulatory release sites.

## Resource availability

### Lead contact

Requests for further information and resources should be directed to and will be fulfilled by the lead contact, Yajun Zhang (yz6k@virginia.edu).

### Materials availability

This study did not generate new unique reagents. Information about the data collection equipment and data analysis approaches is available from the lead contact upon request.

### Data and code availability

• This study did not generate any new original datasets that require public deposition. The data that support the findings of this study are available from the lead contact upon request.
• This code is available at https://github.com/sharonzhengwenxuan/DecodeNeuroFluo.
• Any additional information required to reanalyze the data reported in this article is available from the lead contact upon request.

## Acknowledgments

We thank members of Julius J. Zhu’s, B Jill Venton’s and Mark P Beenhakker’s lab for their suggestions and support. We also thank the Stem Cell Core Facility at the University of Virginia for providing the cell line. This work was funded by 10.13039/100000002NIH R01NS121014 (B Jill Venton), 10.13039/100000002NIH R01NS131670, RF1NS131762 (Mark P Beenhakker) from National Institutes of Health, United States; VADC Basic Science Pilot Funding (to S.G.) from Virginia Alzheimer’s Disease Center, UVA Brain Institute, United States; 10.13039/100000957Alzheimer’s Association Research Fellowship AARF-19-619387 (to P.Z.) from the Alzheimer’s Association, United States.

## Author contributions

W.S.Z., P.Z., and Y.Z. conceived the concept; W.S.Z. developed MATLAB-based image analysis program and analyzed data with Y.Z.; W.S.Z., S.G., P.Z., and Y.Z. performed molecular biology experiments and collected imaging data; W.S.Z. and Y.Z. wrote the manuscript with input from all other coauthors.

## Declaration of interests

The authors declare no competing interests.

## STAR★Methods

### Key resources table

**Table:** 

REAGENT or RESOURCE	SOURCE	IDENTIFIER
**Antibodies**		

Goat anti-serotonin	Abcam	Cat #ab66047; RRID: AB_1142794
Mouse anti-βIII-tubulin (Tuj1)	Bio-Techne	Cat #MAB1195; RRID: AB_357520
Anti-goat Alexa Fluor 488	Abcam	Cat #ab150133
Anti-mouse Alexa Fluor 594	Abcam	Cat #ab150128

**Bacterial and virus strains**		

Sindbis-GRAB_5HT1.0_	This paper	N/A
Sindbis-GRAB_OT1.0_	This paper	N/A
Sindbis-iGluSnFR	This paper	N/A
Sindbis-iGluSnFR3	This paper	N/A
Sindbis-GRAB_NE1m_	This paper	N/A
Sindbis-GRAB_DA2m_	This paper	N/A
Sindbis-GRAB_HA1m_	This paper	N/A
Sindbis-OxLight	This paper	N/A
Sindbis-iAChSnFR	This paper	N/A
DH5α Competent Cells	Invitrogen	Cat #18265017

**Chemicals, peptides, and recombinant proteins**		

Tetrodotoxin (TTX)	Abcam	Cat #ab120055
Serotonin hydrochloride	Tocris	Cat #3547
CLA IdentiFiler™ Plus PCR Amplification Kit	Thermo Fisher	Cat# A47624

**Experimental models: Cell lines**		

Primary human dermal fibroblasts (serotonergic)	Stem Cell Core Facility, UVA	S4FC3

**Experimental models: Organisms/strains**		

Mouse: C57BL/6J	Jackson Laboratory	RRID: IMSR_JAX:000664
Rat: Sprague Dawley	Charles River Laboratories	RRID: RGD_737891

**Recombinant DNA**		

pSin-iAChSnFR	This paper	https://www.addgene.org/140643/
pSin-GRAB_5HT1.0_	This paper	https://www.addgene.org/172882/
pSin-GRAB_NE1m_	This paper	https://www.addgene.org/172883/
Sindbis-GRAB_OT1.0_	This paper	N/A
Sindbis-iGluSnFR	This paper	N/A
Sindbis-iGluSnFR3	This paper	N/A
Sindbis-GRAB_DA2m_	This paper	N/A
Sindbis-GRAB_HA1m_	This paper	N/A
Sindbis-OxLight	This paper	N/A

**Software and algorithms**		

IGOR Pro 6	WaveMetrics	https://www.wavemetrics.com
ImageJ	Fiji	https://imagej.net/software/fiji/
MATLAB R2024a	Mathworks	https://www.mathworks.com/products/matlab.html
DecodeNeuroFluo (Imaging analysis)	Github	https://github.com/sharonzhengwenxuan/DecodeNeuroFluo
Prism	Graphpad	https://www.graphpad.com/
bioRender	BioRender	https://www.biorender.com/
HCImage	Hamamatsu Photonics	https://www.hamamatsu.com/
SigmaPlot 10.0	Systat	https://systatsoftware.com/

**Other**		

23 nm green GATTA beads	GATTAquant	http://www.gattaquant.com/products/gatta-beads.html
Hamamatsu ORCA FLASH4.0 camera	Hamamatsu Photonics	https://www.hamamatsu.com/
460-nm ultrahigh-power low-noise LED	Prizmatix	https://www.prizmatix.com/
Bipolar electrode	FHC	https://www.fh-co.com/

### Experimental model and study participant details

#### Animal and human preparations

All experiments were performed using mice (C57BL/6J) and rats (Sprague Dawley) of both sexes, and no significant sex-dependent differences were observed. Animals were housed at the University of Virginia in temperature-regulated rooms under a 12-h light/dark cycle and maintained in family or paired housing with unrestricted access to food and water. Experimental protocols involving animals and human-derived cells were reviewed and approved by the Animal Care & Use Committee of the University of Virginia and conducted in compliance with NIH guidelines.

Primary human dermal fibroblasts (S4FC3; inducible to serotonergic neurons, hereafter referred to as S4F) were obtained from the Stem Cell Core Facility at the University of Virginia. This line was originally generated in the laboratory of Fred H. Gauge by viral transduction of inducible constructs encoding ASCL1, NGN2, NKX2.2, FEV, GATA2, and LMX1B and was established from skin biopsies from de-identified healthy human donors were transduced to neuron-competent fibroblasts using a previously established protocol.^13^ Serotonergic fibroblasts, capable of expressing serotonergic and neural transcription factors, were seeded on tissue culture grade plastic petri dishes/slides/plates and, after 24 h, the medium was changed to Neural induction media over 3 weeks. The induced neurons (iNs) were then switched to neural maturation medium to mature for 4–8 weeks. Cell identity was confirmed by short tandem repeat (STR) profiling. Reproducibility of lineage conversion was assessed across independent fibroblast lines, and inducible transgene expression was validated by doxycycline-dependent activation. Cell lines were routinely tested for mycoplasma contamination using a PCR-based assay, and all cultures were confirmed to be mycoplasma-free prior to use. Experiments were independently repeated four times. All procedures for animal and human-derived cell line experiments were performed following protocols approved by the Animal Care & Use Committee of the University of Virginia and in accordance with US National Institutes of Health guidelines.

#### Acute brain slice preparations

Brain slices were prepared from animals aged P25–60 following deep anesthesia with xylazine–ketamine and rapid decapitation. Data were collected from at least 5 animals per condition, with exact sample sizes reported in the corresponding figure legends. Brains were immediately transferred to ice-cold (0−4°C), oxygenated aCSF containing (in mM): 125 NaCl, 2.5 KCl, 1.25 NaH_2_PO_4_, 25 NaHCO_3_, 1 MgCl_2_, 25 dextrose, and 2 CaCl_2_, pH 7.4. Target regions including LGN, DR, rat DG, BLA, CPu, PVN, NAcSh, and VTA were sectioned directly from the brain block into 400-μm slices using a microslicer (Ted Pella Inc.). Slices were incubated in oxygenated physiological solution at 37.0 ± 0.5 °C for 30–60 min before imaging.

During experiments, slices were maintained in a submerged recording configuration, stabilized with a nylon mesh affixed to a platinum ring, and continuously perfused with oxygenated aCSF. Bath temperature was maintained at 34.0 ± 0.5 °C, and the solution exchange half-time was approximately 6 s. Pharmacological agents were delivered via puff or bath application.

### Method details

#### Viral constructs and delivery strategy

Genetically encoded glutamate, acetylcholine, monoamine, and neuropeptide fluorescent sensors were sub-cloned into Sindbis constructs and viral particles were produced as described in our previous studies.^35^ In brief, fluorescent sensors and their variants were sub-cloned into Sindbis viral vector pSinRep5 with the cloning sites XbaI and SphI.

GEIs were expressed as previously reported.^35^^,^^36^^,^^37^^,^^38^ P25−60 animals were initially anesthetized by an intraperitoneal injection of ketamine and xylazine (10 and 2 mg/kg, respectively), and then placed in a stereotaxic frame. A glass pipette was used to penetrate the LGN (AP: −2.5, ML: ±2.2, DV: −3.0), DR (AP: −4.36, ML: 0, DV: −3.25), rat DG (AP: −4.0, ML: ±1.5, DV: −4.0), BLA (AP: −1.6, ML: ±3.0, DV: −4.8), CPu (AP: +0.5, ML: ±2.0, DV: −3.0), PVN (AP: −0.6, ML: ±0.3, DV: −4.6), VTA (AP: −3.3, ML: ±0.4, DV: −4.3) or NAcSh (AP: +1.5, ML: ±0.7, DV: −4.4) according to stereotaxic coordinates, to deliver ∼50 nL of Sindbis or lentiviral solution by pressure injection to infect neurons, astrocytes, or non-neuronal cells.

Human induced neurons were infected by including ∼50 nL of viral solution in the culture media. Experiments were typically performed within 18 ± 4 h after Sindbis viral infection or 1−2 weeks after lentiviral infection.

#### Immunostaining of human serotonergic neurons

Human fibroblast-derived serotonergic neurons were fixed with 4% paraformaldehyde for 30 min at room temperature (RT). Following fixation, cells were washed three times with 1× PB, followed by one wash with 1× PBST (PBS containing 0.1% Tween 20). Cells were then permeabilized with 0.3% Triton X-100 in 1× PBS for 10 min at RT. After permeabilization, cells were blocked with a blocking solution containing 2% bovine serum albumin, 1% fish skin gelatin, 0.02% saponin, and 15% horse serum in 1× PBS. Primary antibody incubation was performed overnight at 4°C using goat anti-serotonin and mouse anti-βIII-tubulin. The following day, cells were incubated for 2 h at RT with secondary antibodies: anti-goat Alexa Fluor 488 (1:500) and anti-mouse Alexa Fluor 594 (1:500). Nuclei were counterstained with 4′,6 -diamidino-2-phenylindole (DAPI) for 5 min at RT. Finally, immunostained cells were washed with PBS and mounted for imaging. Experiments were repeated in three independent experimental replicates using serotonergic neurons from matched-passage S4F fibroblast cultures. Each replicate corresponded to an independent culture well.

#### Fluorescence imaging

To minimize drift and fluctuating movements in imaging, which is vital for high-resolution visualization of the transmitter release-induced fluorescence responses,^11^ a stable recording/stimulating and imaging setup was used to carry out all imaging experiments. Wide-field epifluorescence imaging was performed using a Hamamatsu ORCA FLASH4.0 camera (Hamamatsu Photonics, Japan), and fluorescent sensor expressing cells in acutely prepared tissue slices were excited by a 460-nm ultrahigh-power low-noise LED (Prizmatix, Givat-Shmuel, Israel).^35^

The fluorescence signals were collected with an Olympus 40× water-immersion objective (numerical aperture 0.8). The microscopic point-spread function on our image setup was measured with 23 nm fluorescent beads (Figure S2). Image acquisition was performed at sampling rates at 10 Hz by FLASH4.0 camera. To synchronize image capture with electrical stimulation, the camera was set to external trigger mode and triggered by a custom-written IGOR Pro 6 program-based software PEPOI. Glutamatergic, monoaminergic, or neuropeptidergic fibers in tissue slices were stimulated with a bipolar electrode (CE3C65) placed ∼50−200 μm from imaged cells. A single or a train of voltage pulses (400 μs, up to 10 V) was applied to evoke transmitter release.

For puff application experiments, 5-HT dissolved in aCSF was pressure-applied through a glass micropipette (tip diameter ∼10 μm) positioned ∼50–100 μm from imaged cells. Puff pulses were delivered using a pneumatic pressure device (Picospritzer, Parker Hannifin) at 2 psi for 10 s to ensure a stable local concentration.

### Quantification and statistical analysis

#### Imaging analysis

The fluorescence *Δ*F/F_0_ responses were analyzed with code created using MATLAB R2024a with Image Processing Toolbox (Mathworks) and comprised five major algorithmic procedures, including alignment, deconvolution, baseline adjustment, denoising and background correction. The alignment procedure utilized translational intensity-based automatic registration to align images.^39^ The process transformed the fluorescence images into matrices and computed an image similarity metric value by comparing the moving images to the reference image. The iterative transformation process stopped at a point of diminishing returns or after 200 iterations. The movement score is the average displacement of each pixel in one image to the fixed image, which was computed by using a non-parametric diffeomorphic image registration algorithm.^40^ The adjusted maximum movement displacement scores (AMMSs) are the maximum change of movement score during the stimulation. After the translational alignment, images were corrected for optical blurring by performing Landweber deconvolution^41^ using our empirically obtained point spread function.^10^ The process used an iterative gradient-descent method to minimize the value calculated with the least-squares cost function^42^ and, at the same time, imposed a non-negativity constraint at each iteration. We found that 50 iterations typically achieved a good trade-off between image improvement and noise amplification, with the latter largely overcome by the subsequent denoising procedure. Then, the baseline adjustment procedure compensated for the small fluorescence decay associated with GEI-based imaging by applying a two-term exponential curve to fit on the first control 10-s (typically 100 frames) and last control 10-s (typically 100 frames) image sequence data. Multiplication factors were calculated at individual pixels after independent fitting to produce new flat baselines, which were then applied to the entire image sequences. The denoising procedure minimized the random noise by using locally weighted smoothing and linear or exponential fitting in a pixel-by-pixel manner. Finally, the background correction procedure corrected the background level by uniformly subtracting an average fluorescence value estimated from a non-responsive region adjacent to the cell. Despite variable fluorescence F_0_ across the entire cell membrane surface of neurons due to heterogeneous sensor expression, the *Δ*F/F_0_ responses showed no or weak correlation with the basal fluorescence F_0_, suggesting independence from sensor expression levels and reliability in measuring transmitter concentration (Figure S9). To analyze the spatial variability of the fluorescence response, the image frame corresponding to the maximal response was selected. Regions of interest (ROIs) were defined based on the visible cell area expressing the sensor, excluding surrounding background regions. Pixel values within the ROI were extracted for analysis. Background signal was estimated from adjacent non-cell regions and subtracted uniformly. All pixels within the ROI were included in the analysis without additional thresholding, unless otherwise stated. Spatial heterogeneity was quantified using the coefficient of variation (CV), calculated as the standard deviation divided by the mean of all pixel values within the ROI.

#### Synaptic transmission property analysis

To visualize the individual transmitter releasing synapses and estimate the spatial diffusion extent of transmitters at the postsynaptic side, the electrically evoked maximal *Δ*F/F_0_ responses at individual pixels over time were plotted to create 3D spatial profiles for individual transmitter releasing synapses. For consistent visualization, each cell image was rotated to best display the direction of the 3D peak. Individual transmitter releasing synapses were isolated and identified using a density-based spatial clustering algorithm DBSCAN.^43^ Iterative selection and analysis were performed to isolate and identify individual releasing synapses when they formed overlapping clusters. Pixels with the maximal *Δ*F/F_0_ responses in individual releasing synapses were assumed to be the centers of release. Fluorescence *Δ*F/F_0_ intensity profiles were averaged over multiple exposures, multiple releases and/or multiple directions of transmitter diffusion gradients and fit with a single-exponential decay function. The fitting was performed at the well-isolated releasing synapses and their decay constants were extracted as spatial spread length constants for all transmitters.

To estimate the quantal properties of individual transmitter releases, 20-pulse trains at a low-frequency of 0.1 Hz were used to evoke transmitter release, and failures and releases of *Δ*F/F_0_ events at isolated individual releasing synapses were analyzed using a quantal analysis approach,^14^ which yielded the vesicle quantal size, quantal content, and release probability. To estimate the other fundamental presynaptic release properties, 1,920-pulse trains at high frequency of 16 Hz were employed to exhaust transmitter release, and cumulative release against stimulus number plots were fit with the previously established synaptic transmission model,^15^ which revealed the refilling rate and vesicular pool sizes. To isolate the individual pulse-evoked *Δ*F/F_0_ responses evoked by 16 Hz pulse trains, the effect caused by slow sensor fluorescence decay was mathematically minimized using the measured sensor fluorescence decay constants. Increasing stimulation intensity or frequency affected certain synaptic properties but not others.^10^^,^^11^ Thus, we used a minimum stimulation intensity (i.e., 5−10 V) ideal for resolving different synaptic properties in the experiments.

#### Statistical analysis

Quantitative data are represented as mean ± SEM. Sample sizes were selected to balance statistical power and efficiency, guided by the inverse relationship between variability and the square root of the number of observations, resulting in typical group sizes of approximately 10–25. Hypothesis testing was performed using two-sided nonparametric methods, with Wilcoxon tests applied to paired datasets and Mann–Whitney rank-sum tests used for independent two-group comparisons, and Kruskal–Wallis tests were used for comparisons involving more than two independent groups. Effect sizes are reported as rank-biserial correlation (*r*) for Wilcoxon and Mann–Whitney tests and as epsilon-squared (*ε*^*2*^) for Kruskal–Wallis tests, together with 95% confidence intervals, to quantify the magnitude and uncertainty of observed effects. Statistical significance was defined as follows: ns, not significant *p* > 0.05; ∗*p* < 0.05 with significant difference.
