# Supplementary material for: Optical insights into spatial precision and release heterogeneity of neuromodulatory transmission
Source: iScience. 2026 May 20;29(6):116037. doi: 10.1016/j.isci.2026.116037 (PMC13214266; doi:10.1016/j.isci.2026.116037)
Supplement: Document S1. Figures S1–S9 and Table S1 [file mmc1.pdf]

iScience, Volume *29*

Supplemental information

Optical insights into spatial precision and release heterogeneity of neuromodulatory transmission

W. Sharon Zheng, Smriti Gupta, Peng Zhang, and Yajun Zhang

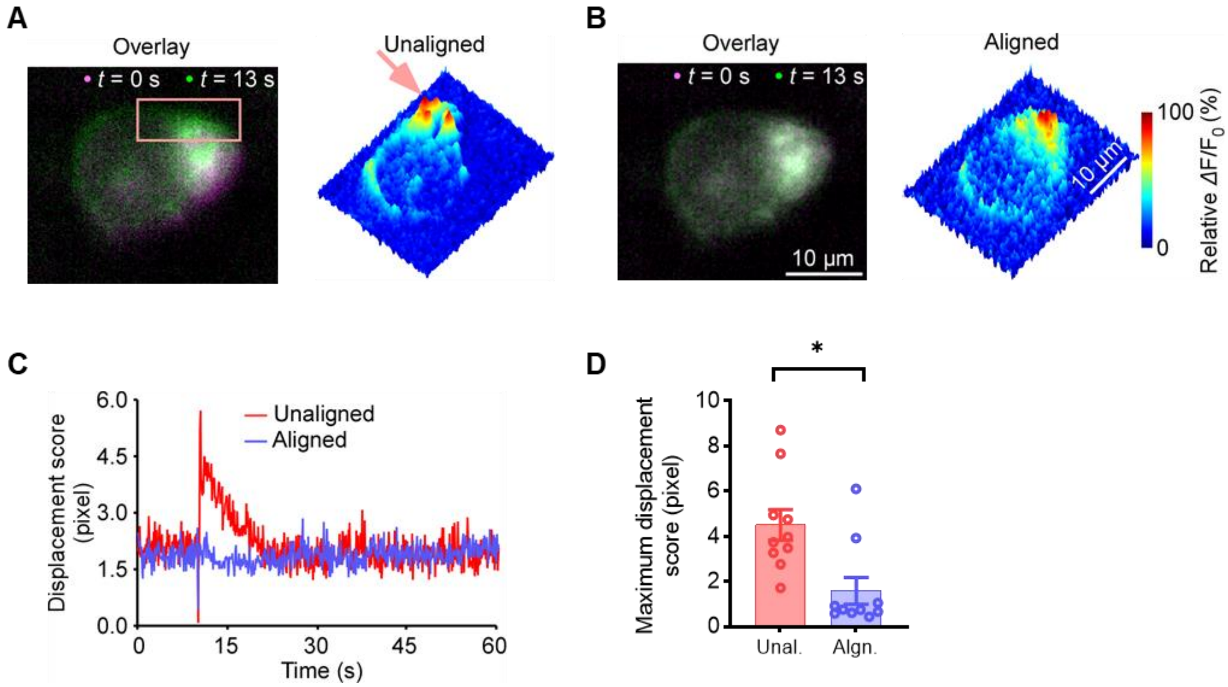

Figure S1. Alignment preserves image faithfulness, related to Figure 1.

(A) Left: Snapshots and overlay of images at two time points, highlighting the prominent mismatch in unaligned control images. Right: 3D spatiotemporal profile of electrically evoked $\Delta F/F_0$ responses, showing spurious peaks caused by image misalignment (example indicated by pink arrow).
(B) Left: Snapshots and overlay of aligned images. Right: 3D spatiotemporal profiling of the aligned neuron. Scale bar, 10 μm.
(C) Displacement score of the geniculate neuron.
(D) Maximal displacement scores before and after alignment (n = 10 neurons). *$p < 0.05$ with significant differences, Wilcoxon test. Data are represented as mean ± SEM.

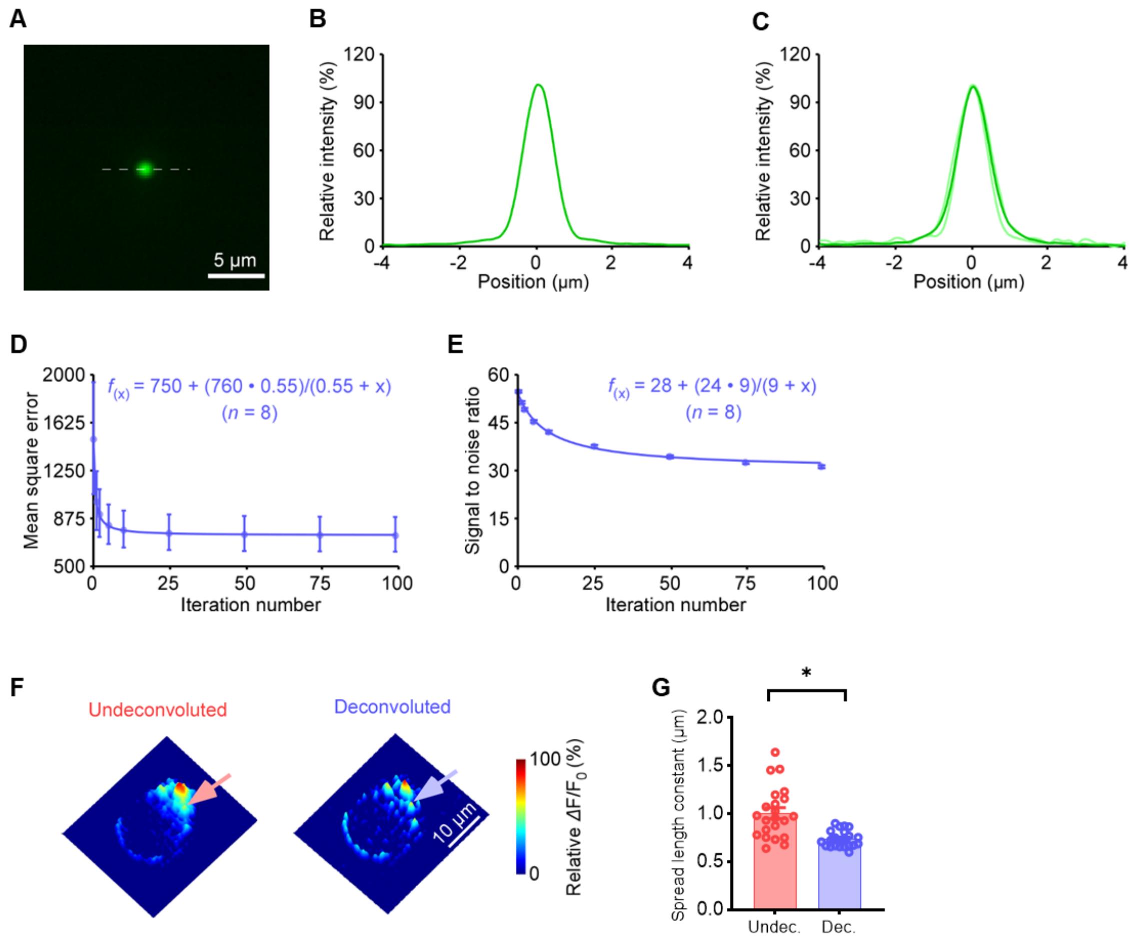

Figure S2. Determination of the PSF for deconvolution, related to Figure 1.

(A) Fluorescence image of a 23-nm green GATTA bead under a 0.8 NA 40x objective. Scale bar, 5 µm.
(B) PSF of the 23-nm green GATTA bead shown in (A).
(C) Individual (light green) and average (dark green) PSFs (n = 10). FWHMs = 0.996 ± 0.021 µm.
(D) Evolution of mean squared error during iterations, fitted with a hyperbolic decay (blue line; r^2 = 0.99).
(E) Evolution of signal-to-noise ratio, also fitted with a hyperbolic decay (blue line; r^2 = 0.99).
(F) 3D profiling of electrically evoked fluorescence $\Delta F/F_0$ responses in the $GRAB_{5HT1.0}$ expressing geniculate neuron under undeconvolved and deconvolved conditions. Scale bar, 10 µm.
(G) Average spread length constants (Undeconvolved: 1.01±0.06 µm; Deconvolved: 0.74±0.02 µm; n = 22 synapses from 8 neurons, Z = -4.042, *p < 0.05 with significant differences, Wilcoxon test. Data are represented as mean ± SEM.

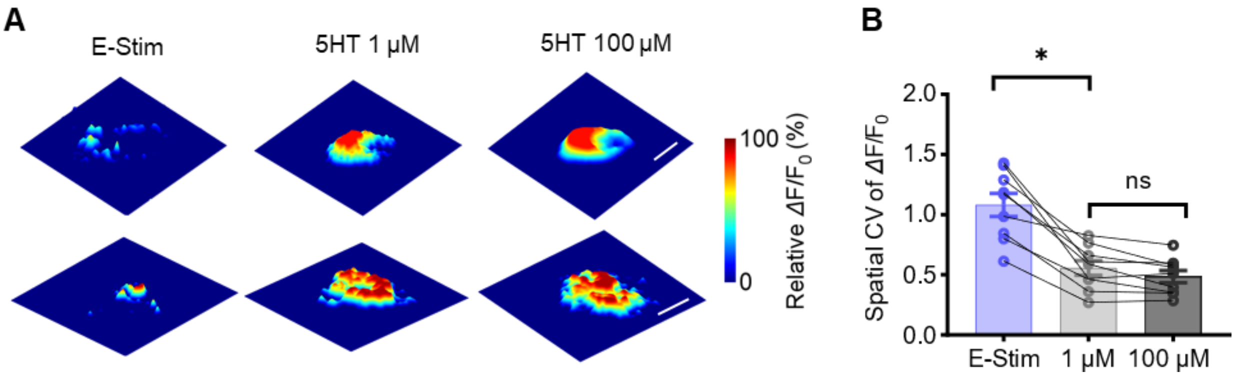

Figure S3. Spatial variability of $GRAB_{5HT1.0}$ response, related to Figure 1.

(A) 3D spatiotemporal profiling of serotonin responses in two example geniculate neurons following electrical stimulation (10 pulses at 16 Hz, 5V) and puff application (1 μM and 100 μM). Scale bar, 5 μm.

(B) Pixel-wise variability of serotonin signals was quantified as the coefficient of variation (CV) across pixel-wise peak $\Delta F/F_0$ values (electrical stimulation: 1.08 ± 0.09; puff 1 μM: 0.56 ± 0.06; puff 100 μM: 0.49 ± 0.05; n = 9 neurons). Electrical stimulation exhibited significantly higher spatial variability compared to both puff conditions (electrical vs. puff 1 μM: Z = 2.60, r = 0.87, *p < 0.01 with significant differences; puff 1 μM vs. puff 100 μM: Z = 1.77, r = 0.59, p = 0.07 with no significant differences, ns. Paired comparisons were performed in the same neuron under identical imaging and analysis conditions using paired Wilcoxon signed-rank tests. Data are represented as mean ± SEM.

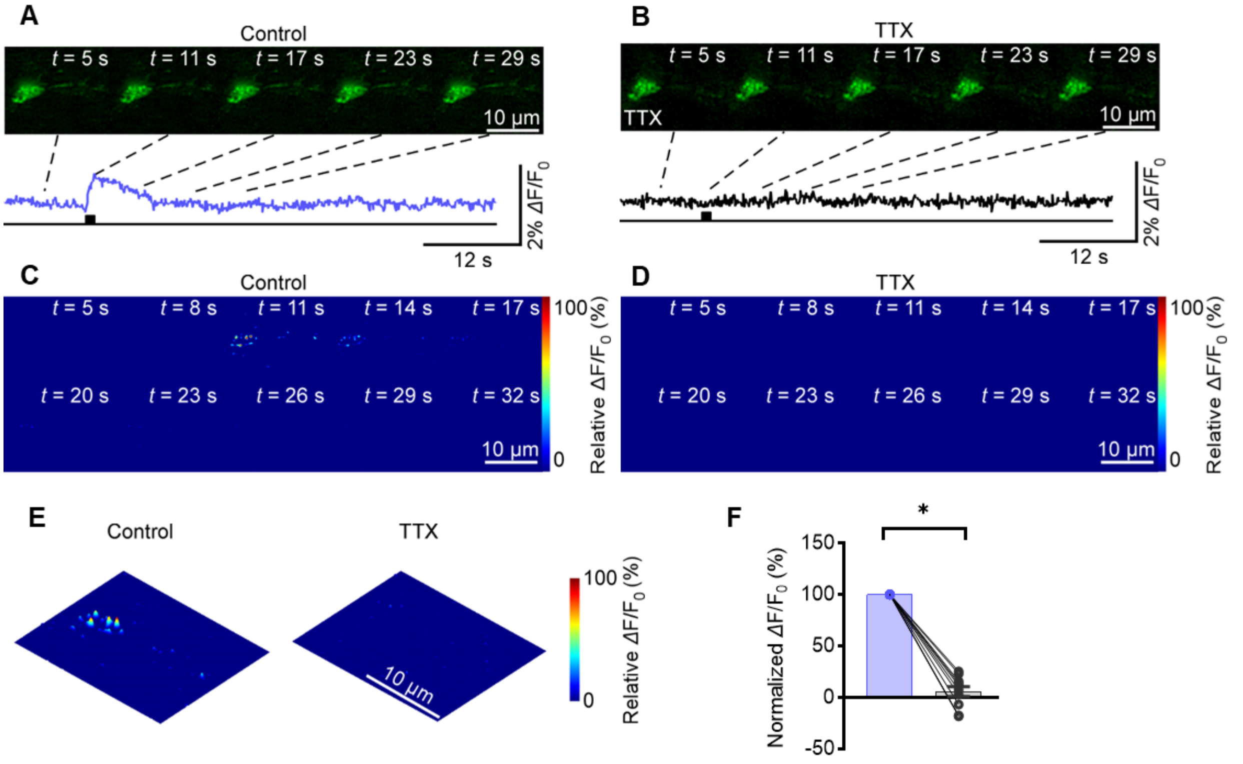

Figure S4. TTX diminishes the evoked $\Delta F/F_0$ responses at GRAB$_{5HT1.0}$ expressing LGN neurons, related to Figure 1.

(A-E) Snapshots (A), (C), heatmaps (B)**,** (D), and 3D spatiotemporal profiling (E) of evoked fluorescence responses under control and 1 μM TTX. Scale bar, 10 μm.
(F) Maximal $\Delta F/F_0$ responses (Control: 100.0 ± 0.0% vs. TTX: 5.7 ± 4.9%; n = 10 neurons, Z = -2.803, *p < 0.01 with significant differences, Wilcoxon test). Data are represented as mean ± SEM.

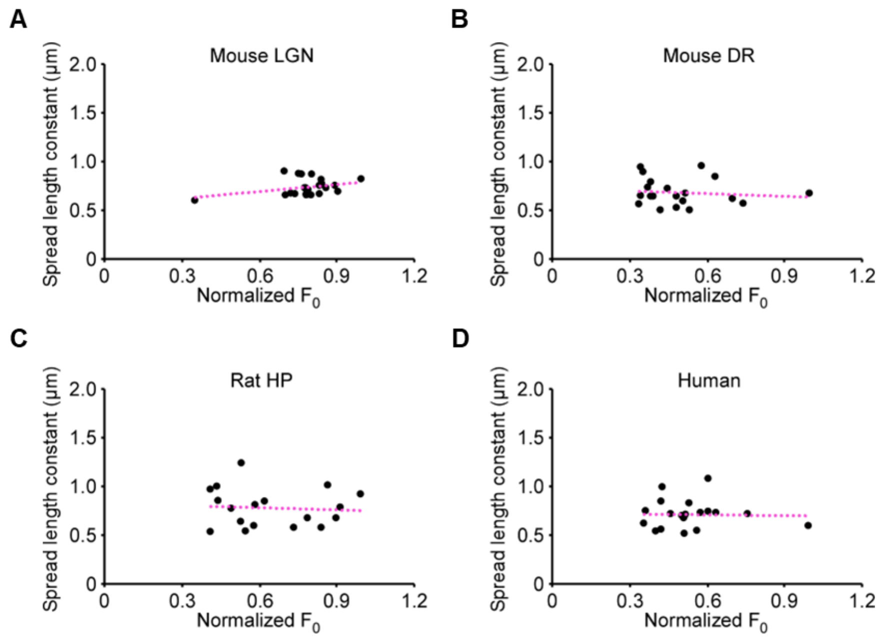

Figure S5. Diffusion length constant is largely independent of sensor expression levels, related to Figure 2.

(A) Plots of spread length constant against normalized F_0 of the $GRAB_{5HT}$ expressing mouse geniculate neurons, fitted by a linear function ($y = 0.23 \cdot x + 0.55$, $r^2 = 0.11$).
(B) Plots of spread length constant against normalized F_0 of the $GRAB_{5HT}$ expressing mouse raphe neurons, fitted by a linear function ($y = -0.093 \cdot x + 0.73$, $r^2 = 0.013$).
(C) Plots of spread length constant against normalized F_0 of the $GRAB_{5HT}$ expressing rat hippocampus neurons, fitted by a linear function ($y = -0.073 \cdot x + 0.81$, $r^2 = 0.0054$).
(D) Plots of spread length constant against normalized F_0 of the $GRAB_{5HT}$ expressing human iPSC-derived neurons, fitted by a linear function ($y = -0.037 \cdot x + 0.72$, $r^2 = 0.0014$).

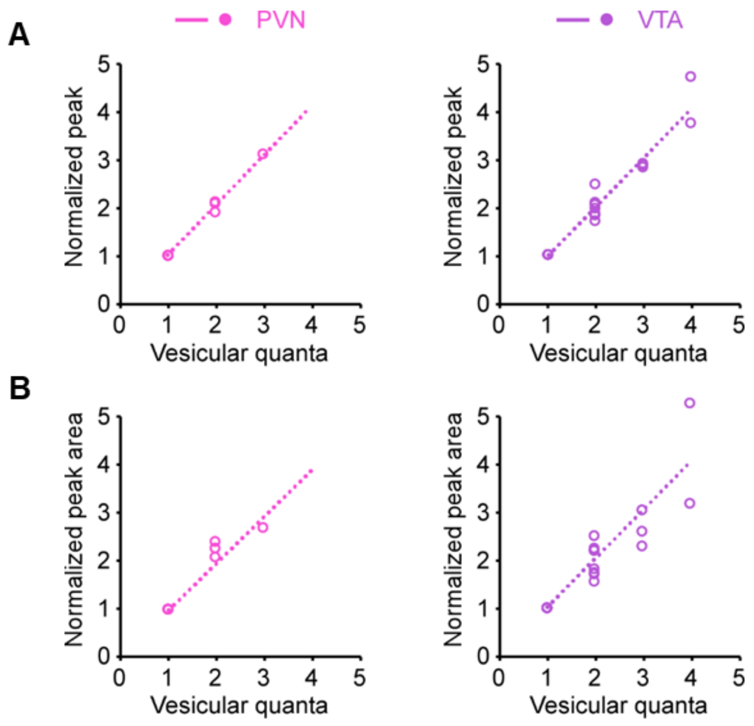

Figure S6. Linear summation of quantal events during multivesicular oxytocin release, related to Figure 5.

(A) Plot of normalized peak amplitudes during multivesicular release, fitted by a linear function (PVN: $y = 1.04 \cdot x$, $r^2 = 1.00$, $n = 10$ neurons; VTA: $y = 1.02 \cdot x$, $r^2 = 1.00$, $n = 10$ neurons).
(B) Plot of normalized peak area of deconvolved single events, fitted by a linear function (PVN: $y = 0.98 \cdot x$, $r^2 = 0.99$, $n = 10$ neurons; VTA: $y = 1.02 \cdot x$, $r^2 = 0.99$, $n = 10$ neurons).

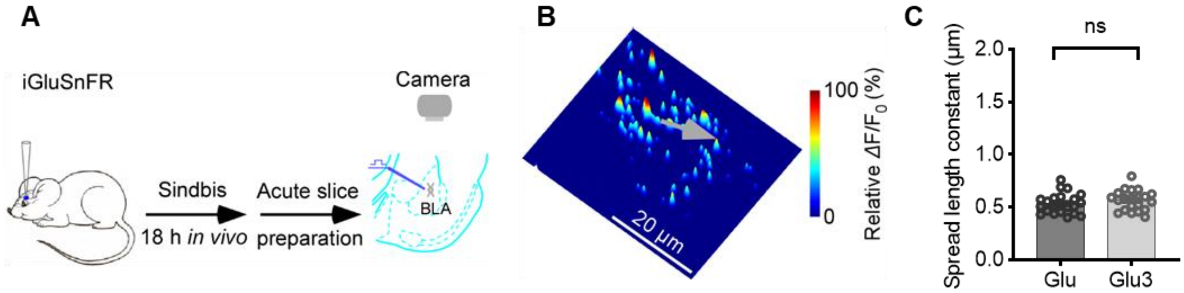

Figure S7. Spatially restricted glutamatergic transmission at amygdalar neurons, related to Figure 6.

(A) Schematic of stimulation-imaging experiment in an *ex vivo* amygdalar preparation expressing iGluSnFR. BLA: the basolateral amygdala.

(B) 3D spatiotemporal profiling of evoked responses in an amygdalar neuron. Dark grey arrow indicates an isolated releasing synapse. Scale bar, 20 μm.

(C) Spread length constants for glutamate using different glutamate sensors (Glu: 0.54 ± 0.02 μm, n = 22 from 10 neurons; Glu3: 0.57 ± 0.03, n = 21 from 8 neurons; U = 183, r = -0.17, 95% CI [-0.53, 0.14], p = 0.25; ns, no significant differences, Mann-Whitney Rank Sum tests). Data are represented as mean ± SEM.

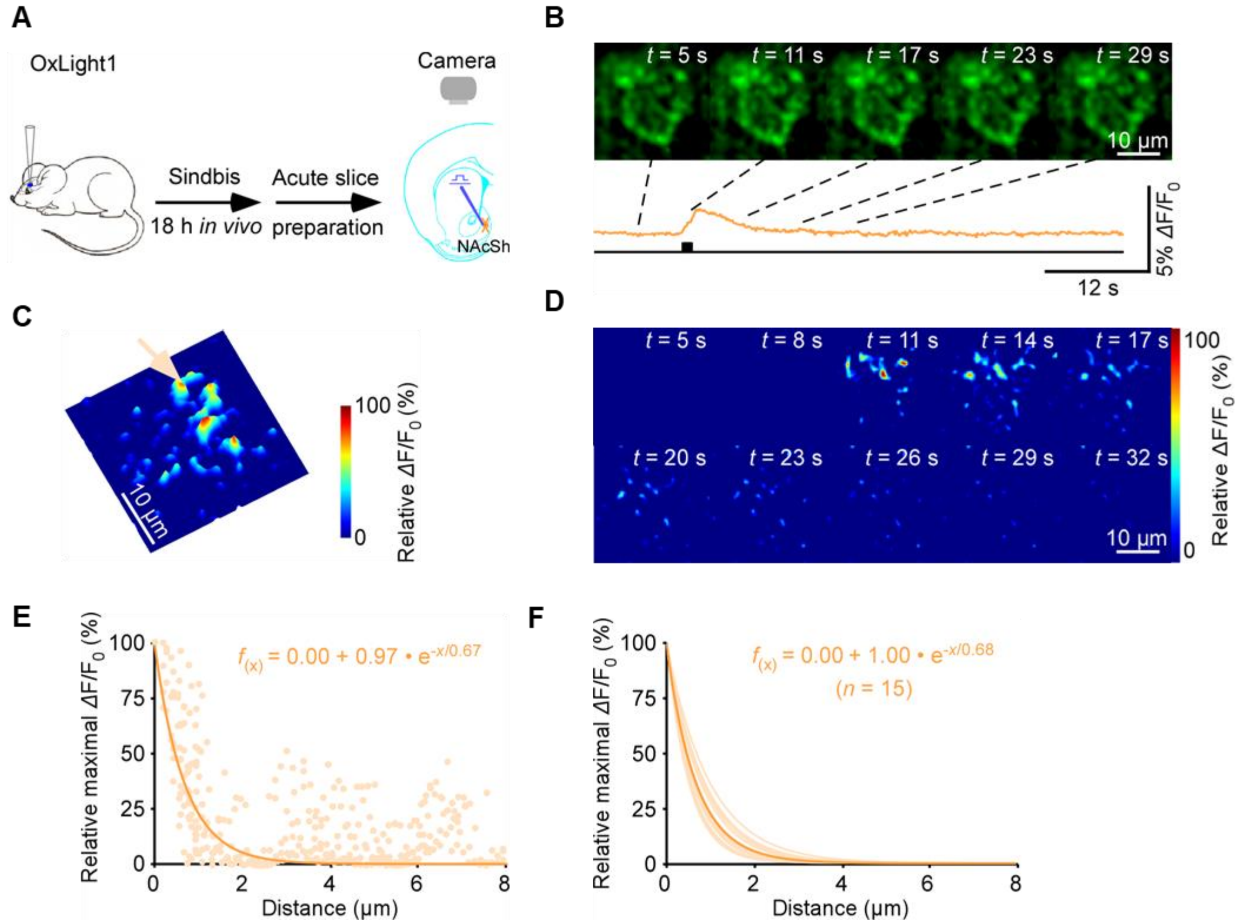

Figure S8. Spatially restricted orexinergic transmission at accumbens neurons, related to Figure 6.
(A) Schematic of stimulation-imaging experiment in an *ex vivo* accumbens preparation. NAcSh: the nucleus accumbens shell.
(B-D) Snapshots (B), 3D spatiotemporal profiling (C), and heatmaps (D) of evoked responses in a nucleus accumbens neuron. Orange arrow indicates an isolated releasing synapse in (C). Scale bar, 10 µm.
(E) Pixel-wise maximal $\Delta F/F_0$ at the isolated releasing synapse. Single-exponential fit (orange line) estimates a spatial spread constant.
(F) Summary of orexin diffusion curves from putative single releasing synapses (spread length constant: 0.68 ± 0.04; n = 15 synapses from 6 neurons, average curve in dark orange).

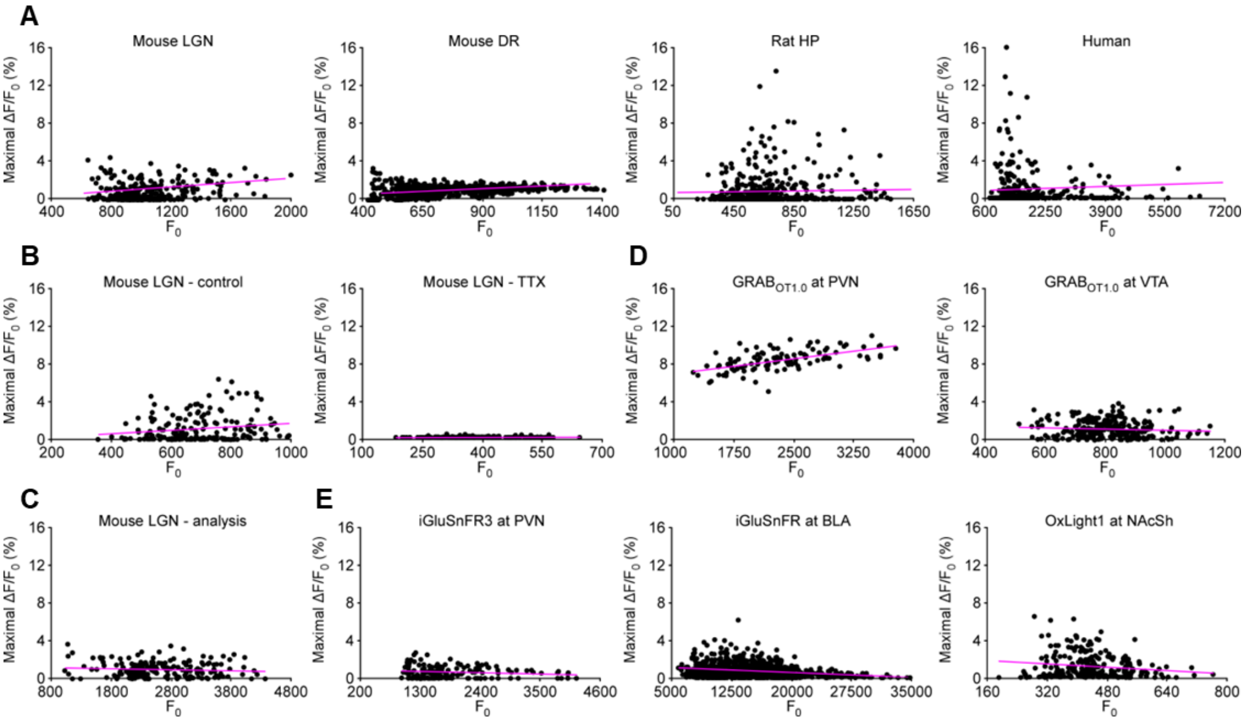

Figure S9. Fluorescence responses are largely independent of sensor expression levels, related to Figure 1-6.

(A) Plots of $\Delta F/F_0$ against F_0 of the $GRAB_{5HT}$ expressing mouse geniculate neurons (Slope of regression line = 0.0000116; Normality test $p < 0.001$; Constant variance test $p = <0.001$; $r^2 = 0.0807$; $F = 640.882$; $n = 7,302$; $p < 0.001$), mouse raphe neurons (Slope of regression line = 0.0000013; Normality test $p < 0.001$; Constant variance test $p < 0.001$; $r^2 = 0.103$; $F = 81.214$; $n = 711$; $p < 0.001$) and rat hippocampus neurons (Slope of regression line = -0.0000044; Normality test $p = 0.2222$; Constant variance test $p = 0.0025$; $r^2 = 0.0426$; $F = 66.3471$; $n = 1,493$; $p < 0.001$), human iPSC-derived (Slope of regression line = 0.00000044; Normality test $p < 0.001$; Constant variance test $p = <0.001$; $r^2 = 0.000843$; $F = 4.518$; $n = 5,354$; $p = 0.034$; Linear regression *t* tests).

(B) Plots of $\Delta F/F_0$ against F_0 of the $GRAB_{5HT}$ expressing mouse geniculate neurons in control (Slope of regression line = 0.0000052; Normality test $p < 0.001$; Constant variance test $p = <0.001$; $r^2 = 0.0029$; $F = 5.1765$; $n = 1,752$; $p < 0.001$), and TTX (Slope of regression line = 0.00000114; Normality test $p < 0.001$; Constant variance test $p = <0.001$; $r^2 = 0.0137$; $F = 23.6859$; $n = 1,705$; $p < 0.001$).

(C) Plots of $\Delta F/F_0$ against F_0 of the $GRAB_{5HT}$ expressing mouse geniculate neurons (Slope of regression line = -0.00000166; Normality test $p < 0.001$; Constant variance test $p = 0.0726$; $r^2 = 0.0224$; $F = 43.1731$; $n = 1885$; $p < 0.001$).

(D) Plot of $\Delta F/F_0$ against F_0 of the $GRAB_{OT1.0}$ expressing mouse paraventricular neuron (Slope of regression line = 0.0000085; Normality test $p = 0.9767$; Constant variance test $p = 0.0561$, $r^2 = 0.180$; $F = 237.344$; $n = 1,038$; $p < 0.001$) and ventral tegmental neuron (Slope of regression line = -0.0000033; Normality test $p <0.001$; Constant variance test $p < 0.001$; $r^2 = 0.0034$; $F = 8.5448$; $n = 2,496$; $p = 0.0035$).

(E) Plot of $\Delta F/F_0$ against F_0 of the iGluSnFR3 expressing mouse paraventricular neuron (Slope of regression line = -0.000000137; Normality test $p < 0.001$; Constant variance test $p < 0.001$; $r^2 = 0.0271$; $F = 39.0578$; $n = 1,405$; $p < 0.001$), iGluSnFR expressing mouse amygdalar neuron (Slope of regression line = -0.00000256; Normality test p < 0.001; Constant variance test p < 0.001; $r^2 = 0.0527$; $F = 54.4167$; $n = 10,106$; $p < 0.001$), and OxLight expressing mouse nucleus accumbens neuron (Slope of regression line = -0.000026; Normality test $p < 0.001$; Constant variance test $p < 0.001$; $r^2 = 0.0220$; $F = 54.4167$; $n = 2,380$; $p < 0.001$).

***Table S1**. **Characteristics** and **comparison of different genetically encoded indicators**.*

Sensors	Kd	Dynamic range	On kinetics	Off kinetics	Membrane targeting	Diffusion constant (µm)
iGluSnFR-A184V	20 µM [S1]	++++	+++++	+++++	+++++	0.54 ± 0.02
iGluSnFR3-V857	200 µM [S2]	+++++	+++++	++++	+++++	0.57 ± 0.03
iAChSnFR	1 µM	++++	+++++	+++++	+++++	0.68 ± 0.02
$GRAB_{NE1m}$	1 µM [S3]	+++	++++	+++++	++++	0.67 ± 0.03
$GRAB_{5HT1.0}$	100 nM [S4]	++++	++++	++++	++++	0.74 ± 0.02
$GRAB_{DA2m}$	100 nM [S5]	+++	++++	++++	++++	0.73 ± 0.05
$GRAB_{HA1m}$	380 nM [S6]	+++	++++	++++	++++	0.75 ± 0.04
$GRAB_{OT1.0}$	3 nM [S7]	+++++	+++++	+++++	++++	0.78 ± 0.03
OxLight	100 nM [S8]	+++	++++	++++	++++	0.68 ± 0.04

Supplemental References

[S1] Marvin, J. S. *et al.* Stability, affinity, and chromatic variants of the glutamate sensor iGluSnFR. *Nat. Methods* **15**, 936–939 (2018).

[S2] Aggarwal, A. *et al.* Glutamate indicators with improved activation kinetics and localization for imaging synaptic transmission. *Nat. Methods* **20**, 925–934 (2023).

[S3] Feng, J. *et al.* A Genetically Encoded Fluorescent Sensor for Rapid and Specific In Vivo Detection of Norepinephrine. *Neuron* **102**, 745-761.e8 (2019).

[S4] Wan, J. *et al.* A genetically encoded sensor for measuring serotonin dynamics. *Nat. Neurosci.* **24**, 746–752 (2021).

[S5] Sun, F. *et al.* Next-generation GRAB sensors for monitoring dopaminergic activity in vivo. *Nat. Methods* **17**, 1156–1166 (2020).

[S6] Dong, H. *et al.* Genetically encoded sensors for measuring histamine release both in vitro and in vivo. *Neuron* **111**, 1564-1576.e6 (2023).

[S7] Qian, T. *et al.* A genetically encoded sensor measures temporal oxytocin release from different neuronal compartments. *Nat. Biotechnol.* **41**, 944–957 (2023).

[S8] Duffet, L. *et al.* A genetically encoded sensor for in vivo imaging of orexin neuropeptides. *Nat. Methods* **19**, 231–241 (2022).
